# Effect of Si on the Impact-Abrasive Wear Behavior of Medium-Carbon Low Alloy Steels with Different Microstructure

**DOI:** 10.3390/ma18245575

**Published:** 2025-12-11

**Authors:** Ziduan Wang, Changhong Cai

**Affiliations:** 1 Brunel London School, North China University of Technology, Beijing 100144, China; wangziduan0816@163.com; 2 School of Mechanical and Materials Engineering, North China University of Technology, Beijing 100144, China; 3 School of Materials Science and Engineering, University of Science and Technology Beijing, Beijing 100083, China; 4 Shiheng Special Steel Group Co., Ltd., Taian 271612, China

**Keywords:** impact-abrasive wear, medium-carbon low-alloy steels, heat treatment, retained austenite, mechanical properties

## Abstract

This work systematically investigated the effect of Si content on the impact-abrasive wear mechanism of medium-carbon low-alloy steels processed through different heat treatment processes, quenching and tempering (QT), austempering, and quenching and partitioning (QP). Three experimental steels with different Si contents were subjected to optimized heat treatment parameters. Microstructural characterization revealed that Si addition significantly enhanced the volume fraction and mechanical stability of retained austenite (RA), refined bainitic and martensitic structures, and suppressed carbide precipitation. The results of mechanical properties demonstrated that austempering yielded the optimal balance of strength, hardness, ductility, and toughness. Impact-abrasive wear tests showed that the 2 B-300 steel exhibited the lowest wear mass loss due to its high work-hardening capacity, deep strain-hardened layer, and low residual tensile stress. In contrast, QT and QP processes resulted in higher wear losses, correlated with high residual tensile stress and reduced RA stability. The above results underscore that Si alloying, combined with appropriate heat treatment, effectively tailors microstructural evolution and residual stress distribution, thereby enhancing impact-abrasive wear resistance for applications in mining and mineral processing equipment. This study provides a comprehensive framework for optimizing Si content and heat treatment parameters to achieve superior wear performance in medium-carbon low-alloy steels.

## 1. Introduction

Impact-abrasive wear represents a prevalent form of material degradation in industries, such as transportation, mineral processing, and metallurgy [1]. Under these conditions, components are subjected to intense impact coupled with rolling and sliding contacts, resulting in surface damage, including plowing, cutting, crack initiation and propagation, and fatigue [1]. This wear mode is a direct cause of failure in metallic components, leading to substantial economic losses in modern industry [2]. Consequently, the advancement of wear-resistant metallic materials has significant technological and economic implications, driving extensive research efforts over decades to mitigate the losses caused by impact-abrasive wear [2]. Although Hadfield steel (high Mn steel) has been widely utilized for its exceptional impact toughness and work-hardening capacity, its limitations in strength and initial hardness restrict its service life in ore crushing and grinding applications [3]. This disadvantage results in unscheduled downtime, material waste, and environmental pollution [4].

Generally, the abrasive wear resistance of steels can be significantly enhanced by improving the matrix strength and surface hardness [5]. Thus, martensitic and bainitic microstructures, which inherently have high strength and hardness, are considered as promising candidate materials [6]. Furthermore, the introduction of a multiphase microstructure containing retained austenite (RA) can yield a synergistic effect, offering an excellent combination of strength and toughness [7]. The formation of tempered martensite with high hardness through quenching and low-temperature tempering (QT) has emerged as a notable strategy for enhancing abrasive resistance. This approach is effective because the resulting microstructure significantly improves resistance to crack propagation during wear [8]. Peng et al. [9] investigated the wear mechanism of 70Mn martensitic steel, and the results suggested that the delamination and fracture resulting from plastic deformation were the primary surface failure mechanism under impact-abrasive wear. Lindroos et al. [10] studied the wear and deformation behavior of wear-resistant steels containing tempered martensite, and demonstrated that the steels had excellent impact resistance. However, for the high-hardness steels consisting of only tempered martensite, the plasticity and impact toughness are frequently compromised, thereby limiting the service life of the resulting wear-resistant products. The bainitic steels containing RA show superior mechanical properties, enabling a favorable balance between strength and toughness [11]. Zhang et al. [12] suggested that bainitic steel had a good impact-abrasive wear resistance and showed a preferable work-hardening ability. Furthermore, a crucial mechanism for enhancing impact-abrasive wear resistance involves the martensitic transformation of film-like RA [13]. This phase transformation from FCC to BCC induces compressive residual stress due to volumetric expansion, which effectively suppresses crack nucleation and propagation [14]. Nevertheless, the inherently low initial hardness of bainitic microstructure may have an adverse effect on impact-abrasive wear resistance. Quenching and partitioning (QP) steel can provide high hardness and strength [15]. Furthermore, when a suitable fraction of fine-scale RA is distributed among martensitic laths, the transformation-induced plasticity (TRIP) effect readily enhances plasticity and toughness due to the improved strain hardening capacity [16]. Additionally, introducing RA into martensite and bainite is an effective strategy to mitigate brittleness—a critical factor governing the service life of equipment subjected to high-speed and high-impact loads [17]. Lu et al. [18] investigated the microstructure, mechanical properties, and impact-abrasive wear behavior of medium-carbon steel treated by the QP process, and showed that the steel had an excellent impact-abrasive wear resistance. However, the influence of the above three processes on the wear resistance in terms of the same steel and its correlation with the resulting microstructure and mechanical properties remains unclear.

Si has an important effect on the wear resistance of steels. Zhang et al. [19] showed that the increase in Si content improved the wear resistance of bainitic steel due to the refined microstructure and enhanced hardness. Nouri et al. [20] investigated the influence of Si on the wear mechanism of hot work tool steel, and showed that the wear resistance increased when the Si content increased from 0.34 wt. % to 0.91 wt. %, but a further increase to 2.26 wt. % showed no significant change. Xie et al. [21] studied the effect of Si on microstructure and wear properties of high vanadium wear-resistant alloy, and demonstrated that when the Si content was 2.14 wt. %, the alloy had excellent comprehensive mechanical properties and wear resistance, resulting from a larger amount of high-hardness pellets and agglomerated VC and a refined nano-bainitic matrix. Lai et al. [22] investigated the influence of Si content on the wear resistance of high chromium cast iron, and the results showed that the increase in Si addition from 0.5 to 1.5 wt. % contributed to the increase in hardness and improvement of abrasive wear resistance, mainly due to the denser secondary carbide formation. Nevertheless, the influence of different Si contents on the microstructure and wear resistance of medium-carbon low-alloy steels remains poorly understood.

In this study, three different heat treatments, including QT, austempering, and QP processes, were applied to medium-carbon low alloy steels with different Si contents to achieve various microstructures. The present work aims to investigate the effect of Si on the microstructure and mechanical properties, and, in particular, the microstructure evolution and its relationship with the impact-abrasive wear mechanism are analyzed in detail. Moreover, the effect of different heat treatment processes on surface residual stress and its relationship with impact wear resistance is investigated. This study provides valuable perspectives for alloy design and heat treatment optimization in medium-carbon low-alloy steels that require desired impact-abrasive wear resistance.

## 2. Experimental Procedure

### 2.1. Materials

The investigation utilized three experimental ingots, synthesized in a vacuum under stringent laboratory controls. Each ingot weighed 50 kg and was alloyed with different concentrations of Si. A homogenization procedure was first applied, wherein the ingots were held at 1200 °C for 4 h. They were then subjected to hot forging within a deformation temperature window of 1150–950 °C, resulting in blanks with cross-sectional dimensions of 120 mm × 32 mm. Finally, the blanks were cooled in air to room temperature (RT). The specific chemical compositions of the steel samples are provided in Table 1. The Si, Mn, Cr, Ti, and P contents were determined by an inductively coupled plasma emission spectrometer (ICP-OES, 725 ES, Agilent Technologies, Inc., Santa Clara, CA, USA) according to GB/T 4336-2016 standard [23]. The C and S contents were determined by a high-frequency infrared carbon/sulfur determinator (CS-600, LECO Corporation, Midland, ON, Canada) according to GB/T 14265-2017 standard [24], and O content was determined by a Nitrogen-Oxygen determinator (TC-436, LECO Corporation, Midland, ON, Canada) according to GB/T 11261-2006 standard [25]. Medium-carbon steels containing martensite or bainite were widely used as wear-resistant materials [2,18]. Numerous studies indicated that medium-high carbon content significantly enhanced the wear resistance of steels, especially used in forged grinding balls in high-impact mills [26,27,28]. The addition of small amounts of Cr and Mn can enhance the hardenability of steels without significantly increasing the cost [29]. The addition of Ti can have an important effect on the grain refinement, precipitation strengthening, and improved wear resistance [30,31]. Si plays a significant role in preventing cementite precipitation, increasing strength and hardness, and improving wear resistance [20,32]. This work mainly investigated the effect of Si on the wear behavior. Additionally, the carbon equivalent (CE) in medium-carbon low-alloy steels generally affects hardness, strength, and abrasive wear resistance [26]. Thus, Table 1 lists the CE of the three steels according to the formulae in the reference [33].

### 2.2. Heat Treatments

In this work, three different kinds of heat treatments commonly used in wear-resistant steels were employed, including QT, austempering, and QP. The influence of Si contents on the wear resistance mechanisms of the steels under different heat treatments was investigated. These heat treatment parameters were selected based on dilatometry experiments, theoretical calculations, and corresponding studies [2,18]. The dilatometry specimens with the dimensions of Φ 3 mm × 10 mm (diameter × height) were machined from the quarter of the thickness and width of the forging blanks. These specimens were heat-treated in a dilatometer (L78 RITA, LINSEIS GmbH, Selb, Germany), where induction heating was carried out at a low pressure of 10^−4^ mbar, and the cooling process was achieved through a nitrogen gas flow. An S-type thermocouple was fixed by spot welding at the center of the sample surface to monitor and adjust the temperature during the heat treatment [34]. The austenitization temperature was first established by means of dilatometry experiments. Subsequently, the martensite start temperature (M_s_) was determined. Finally, the specific parameters for the three heat treatments were selected based on further dilatometry analysis.

### 2.3. Characterization

The microstructural analysis was performed on the longitudinal section parallel to the forging direction. For this purpose, the samples in both the initial and heat-treated conditions were prepared using standard metallographic techniques, including grinding, polishing, and etching with a 4 vol. % nital solution. The resulting microstructure was then examined using a field emission scanning electron microscope (FE-SEM, Zeiss SIGMA 500, Zeiss Ltd., Braunschweig, Germany).

The volume fraction of RA (*f*_RA_) and its carbon content (*w*_C_) were determined by X-ray diffraction (XRD) analysis. The experiments were conducted on a Rigaku SmartLab diffractometer (Tokyo, Japan) equipped with a Cu-*K_α_* radiation source, operating at 40 kV and 150 mA. XRD patterns were acquired using a step size of 0.005° and a scanning speed of 1°·min^−1^. The *f*_RA_ value was quantified based on the methodology detailed in reference [35]. Prior to testing, the specimen surfaces were ground and subsequently electro-polished in a 5 vol. % perchloric acid-alcohol solution at 38 V for 15 s to eliminate surface residual stress.

The tensile specimens were fabricated from the quarter-thickness and quarter-width location of the forging blanks, with their long axis aligned parallel to the forging direction. The specimen geometry featured a gauge length of 15 mm, a width of 5 mm, and a thickness of 2 mm. Quasi-static tensile tests were performed on a universal testing machine (EM6.304 L, Shenzhen Tesmart Instrument and Equipment Co., Ltd., Shenzhen, China) at a constant strain rate of 0.001 s^−1^, in accordance with the GB/T 228.1-2021 standard [36]. A contact extensometer (Epsilon 3442, Epsilon Technology, Jackson, WY, USA) was employed for strain measurement at RT. The reported tensile properties for each steel and heat treatment condition represent the average of three valid tests. Furthermore, Charpy V-notch impact tests were conducted at RT using an instrumented pendulum impact tester, following the GB/T 229-2020 standard [37]. The standard impact specimen dimensions were 7.5 × 10 × 55 mm^3^, oriented with the V-notch perpendicular to the forging direction. The Charpy impact energy (CIE) values were also determined as the average of three valid tests per condition.

Hardness tests were performed on a Vickers tester (HM-112, Mitutoyo Ltd., Kanagawa, Japan) with a diamond pyramid-shaped indenter and measured with a load of 500 g and a holding time of 15 s. The hardness value was determined by averaging the ten valid measurements.

### 2.4. Measurement of Surface Residual Stress

An X-ray diffractometer (X-RAYBOT, MRX-RAYS, Brumath, France) was performed to measure the surface residual stress for different heat treatments according to the iso-inclination method in the sin^2^*ψ* approach from GB/T 7704-2017 standard [38]. The influence of the residual stress under different heat treatments on the wear resistance was investigated. For the steel samples, the Cr-*K_α_* radiation and {211} diffraction peak are generally used [39,40]. During the experiments, the X-ray tube voltage and current were set to 20 kV and 1 mA, respectively. Thirteen different tilt angles (*ψ*) from −40° to 40° were used. In addition, the minimum resolution is 0.01°. The positions of the {211} diffraction peaks were evaluated. Additionally, then the X-ray elastic constants 1/2 *S*_2_ = 5.8 × 10^−6^ MPa^−1^ and −*S*_1_ = 1.27 × 10^−6^ MPa^−1^ were used in stress calculations [41]. The samples after heat treatments were directly electro-polished by an electrolytic polishing machine (LectroPol-5, Struers, Ballerup, Denmark) in order to eliminate the effect of the grinding process on the residual stress measurement, and the polished depth and diameter were ~50 μm and 5 mm, respectively. The residual stress analysis was performed on the specimens of the same dimensions and test surface as those used for the impact wear evaluations.

### 2.5. Impact-Abrasive Wear Tests

Impact-abrasive wear tests for different heat treatments were performed on an impact-abrasive machine (MLD-10, Xuanhua Kehua Testing Machine Manufacturing Co., Ltd., Zhangjiakou, China) at RT. The upper specimens after heat treatments used for the wear tests were cut into 10 × 10 × 30 mm^3^ with the wear surface ground using 2000-grit sandpaper, and the height parallel to that of the forging blank. The lower specimen was made of 45 steel with a hardness of 750 HV, and its rotary speed was 200 rpm. The abrasive material was quartz sand with a size of 0.1–4 mm, and the flow rate was 50 kg/h. The impact frequency of the upper sample was 150 times/min. The impact-abrasive wear tests were conducted under the impact energy of 4 J, and three parallel tests were performed for each heat treatment. The wear mass loss was measured per 0.5 h following ultrasonic cleaning in alcohol. After the impact wear test, the worn surface of the sample was observed using FE-SEM to analyze the wear mechanisms under different heat treatments. To investigate the thickness of the work-hardening layer after impact-abrasive wear, the samples were bisected along the impact direction, and the microhardness tests were conducted on the longitudinal cross-section from the worn surface to a specific depth. The microhardness values were the average of ten parallel tests.

## 3. Results

### 3.1. Determination of Heat Treatment Parameters

**Figure 1:**
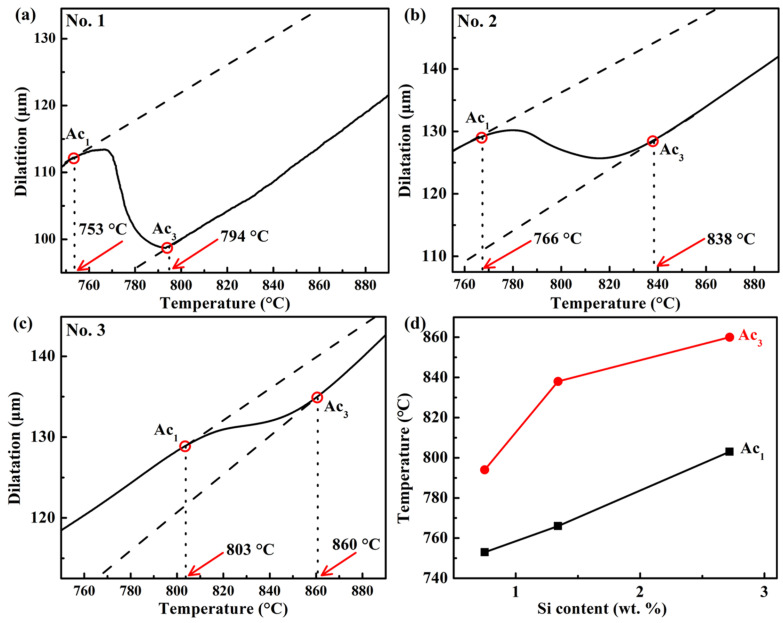
Experimental dilatation curves during heating of the three steels: (**a**) No. 1, (**b**) No. 2, and (**c**) No. 3, and (**d**) Ac_1_ and Ac_3_ as a function of Si content.

**Figure 2:**
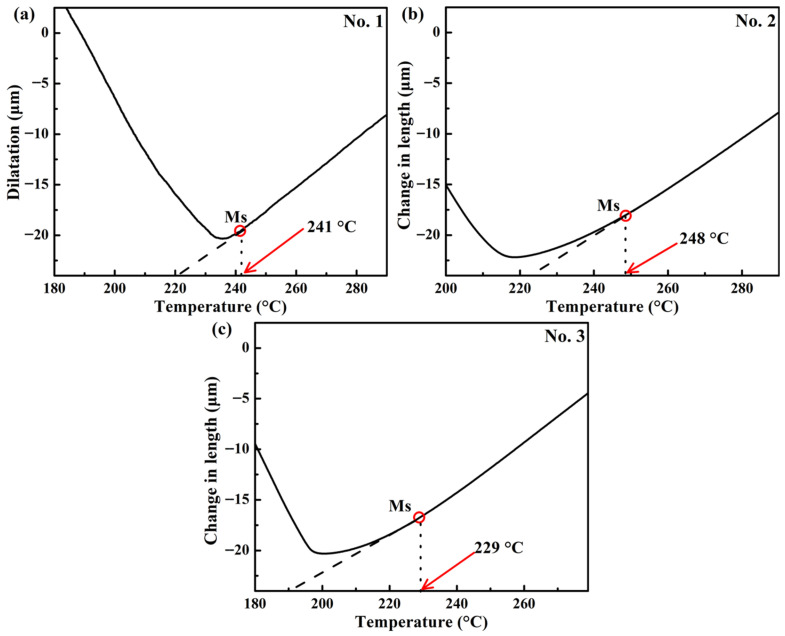
Experimental dilatation curves during cooling of the three steels performed at 900 °C for 30 min: (**a**) No. 1, (**b**) No. 2, and (**c**) No. 3.

The parameters for different heat treatments were determined based on dilatometry experiments, theoretical calculations, and previous studies [2,18]. Firstly, the dilatometry experiments were carried out to determine the critical phase transformation temperatures Ac_1_ and Ac_3_. The specimens were heated to 950 °C at 10 °C/s, followed by quenching to RT at 50 °C/s. Figure 1 shows the experimental dilatometry results of the samples No. 1, No. 2, and No. 3. It can be seen that Ac_1_ and Ac_3_ both increase with the increase in Si content, which is consistent with the results of the references [42,43]. Based on the above experimental results, the austenitizing temperature was selected as 900 °C for the three heat treatment processes applied to the steels with varying Si contents to ensure consistency. According to the previous work [18], the austenitizing time was set as 30 min. Secondly, the Ms was determined according to the following dilatometry experiments. The specimens were heated to 900 °C for 30 min at 10 °C/s, followed by quenching to RT at 50 °C/s, and the results are shown in Figure 2. The Ms of the No. 1 and No. 2 steels is almost the same, and the Ms of the No. 3 steel is lower than that of the others, which is in agreement with the results of the references [44,45]. Si can decrease in Ms and enhance austenite stability.

For the QT process, according to the results of the references [2,46,47,48], the tempering temperatures were set as 200 °C, 250 °C, and 300 °C, with the tempering time of 90 min. They are referred to as ‘1/2/3-QT-200’, ‘1/2/3-QT-250’, and ‘1/2/3-QT-300’.

**Figure 3:**
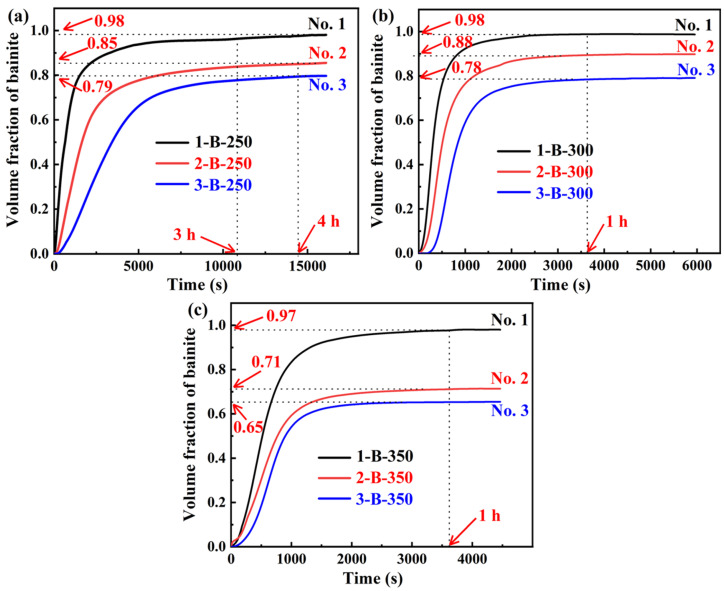
Experimentally volume fraction of bainite as a function of isothermal holding time at different temperatures: (**a**) 250 °C, (**b**) 300 °C, and (**c**) 350 °C.

For the austempering process, the isothermal holding temperatures were selected between Ms and Bs (bainite start temperature), where bainite transformation occurs without martensite formation. In addition, to ensure a good combination of hardness, strength, ductility, and toughness, and with reference to the relevant studies [49,50,51], the isothermal temperatures were set as 250 °C, 300 °C, and 350 °C. The isothermal time was chosen according to the results of dilatometry experiments. The specimens of the three steels were heated to 900 °C for 90 min, followed by quenching to 250 °C, 300 °C, and 350 °C, and isothermally held for a specific duration, and then quenched to RT. The cooling rate was 50 °C/s. The volume fraction of bainite as a function of holding time was determined by applying the lever rule on the dilation data [52] and the bainite fraction at the endpoint obtained by XRD. Figure 3 shows the volume fraction of bainite as a function of holding time at different isothermal temperatures. It can be seen that when the isothermal temperature is 250 °C, the No. 1 steel requires 3 h to substantially complete the bainitic transformation, whereas the No. 2 and No. 3 steels require a longer duration of 4 h. When the isothermal temperatures are 300 °C and 350 °C, one hour is sufficient for the three steels to substantially complete the transformation. Thus, the isothermal temperatures and the corresponding times for the austempering processes of the three steels were determined. They are referred to as ‘1/2/3 B-250’, ‘1/2/3 B-300’ and ‘1/2/3 B-350’. Furthermore, in Figure 3, the final bainite content decreases with the increase in Si content for each isothermal temperature, and for the No. 2 and No. 3 steels, the final bainite content shows a decreasing trend with the increase in isothermal temperature. The addition of Si can inhibit the precipitation of carbides and promote the enrichment of C in the austenite, which can improve the stability of RA [53]. Moreover, increasing Si content can reduce the bainitic transformation kinetics and cause the incompleteness of bainitic transformation [54].

**Figure 4:**
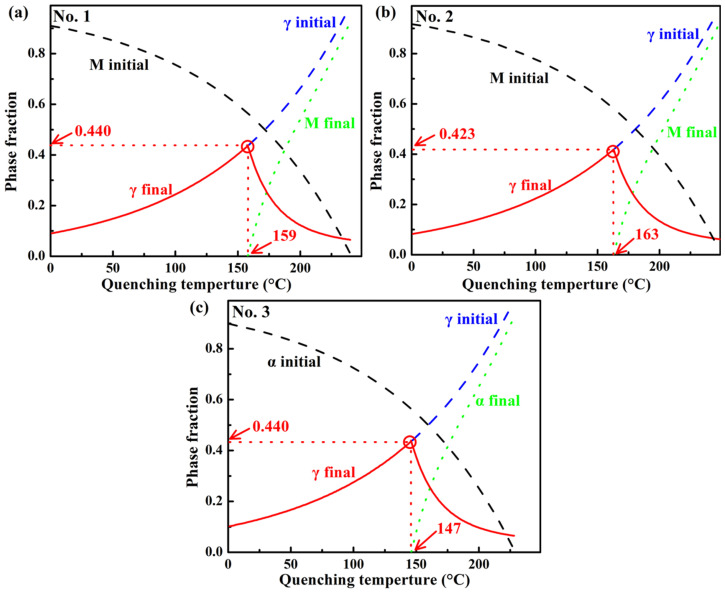
Predicted evolution of microstructural constituents with quenching temperature without consideration of other competing reactions, such as C clustering, C trapping at dislocations, and carbide precipitation, in the investigated three steels: (**a**) No. 1, (**b**) No. 2, and (**c**) No. 3. M is martensite and γ is austenite.

The predictions of the microstructural evolution for the QP process were calculated theoretically based on the Koistinen–Marburger relationship and the two key assumptions: complete carbon partitioning between martensite and austenite and a homogeneous composition of high-temperature austenite [55,56]. The calculated results and the optimized quenching temperatures are shown in Figure 4, and the latter corresponds to the maximum *f*_RA_. It can be seen that Si does not have a significant effect on the maximum *f*_RA_. Based on the fact that the best mechanical properties were not at the maximum *f*_RA_, and the theoretical calculations generally deviated from the experimental results [57,58], in this work, three quenching temperatures (120, 150, and 180 °C) are selected. Additionally, to ensure sufficient C partitioning from martensite to austenite and suppress carbide precipitation and austenite decomposition during the partitioning process, the partitioning temperatures of 300 °C, 350 °C, and 400 °C were selected. The specific heat treatment parameters for the QP process, designated as QP-1, QP-2, etc., are detailed in Table 2.

### 3.2. Mechanical Properties of the Three Steels Treated by Different Heat Treatments

#### 3.2.1. Mechanical Properties of the Three Steels Treated by QT Processes

**Figure 5:**
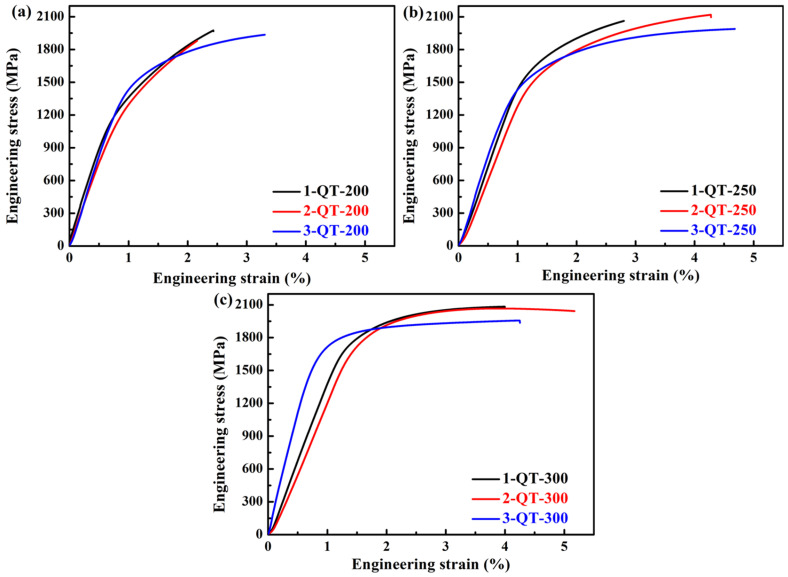
Representative engineering stress–strain curves of the investigated three steels treated by different tempering temperatures for QT processes: (**a**) 200 °C, (**b**) 250 °C, and (**c**) 300 °C.

**Figure 6:**
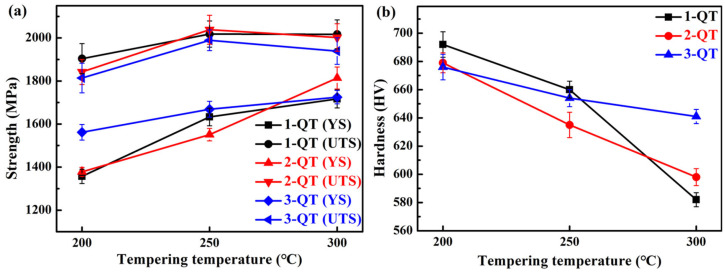
(**a**) YS and UTS and (**b**) hardness with different tempering temperatures of the investigated three steels.

Table 3 and Figure 5 show the mechanical properties (including Young’s modulus (E)) and representative tensile curves of the investigated three steels treated by QT processes, respectively. It can be seen that the yield strength (YS) of the three steels gradually increases with the increase in tempering temperature, and the ultimate tensile strength (UTS) exhibits a trend of initially increasing and then decreasing (see Figure 6a). For example, for the No. 2 steel, the YS increases from 1378 MPa to 1814 MPa when the tempering temperature increases from 200 °C to 300 °C. The increase in strength during tempering mainly results from the *ε*-carbides dispersed in the martensite matrix [59,60], or may be related to the relaxation of internal stress in the martensite structure [61]. Furthermore, during tempering, the dislocation density rapidly decreases, resulting in a low strain hardening rate and thus the decrease in UTS [62]. The total elongation after fracture (TEL) does not change significantly with the increase in tempering temperature, and it is almost under 5%, demonstrating low plasticity. In Figure 6b, the hardness continues to decrease with the increase in tempering temperature for the three steels, mainly due to the recovery softening of martensite and supersaturated carbon dissolving within the martensite [62]. In addition, the maximum and minimum hardnesses are 692 HV and 582 HV of the 1-QT-200 and 1-QT-300 steels, respectively. The steels treated by QT processes have low CIE (<10 J), as shown in Table 3.

#### 3.2.2. Mechanical Properties of the Three Steels Treated by Austempering Processes

**Figure 7:**
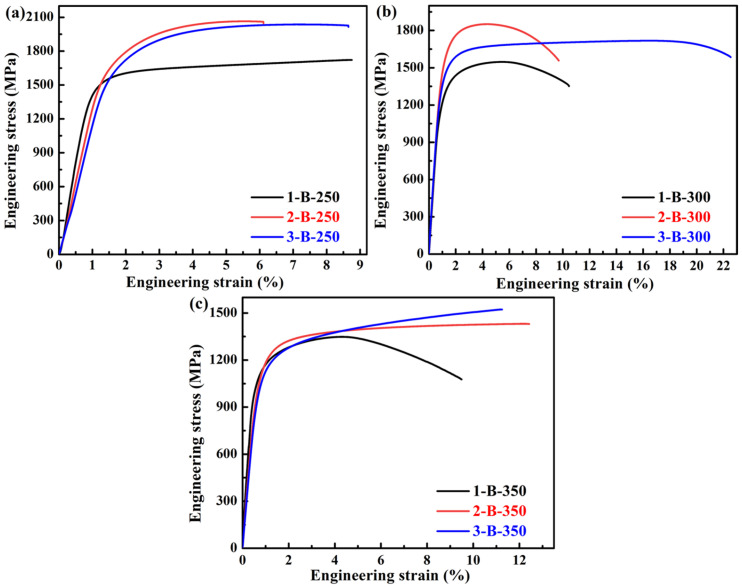
Representative engineering stress–strain curves of the investigated three steels treated by different isothermal temperatures for austempering processes: (**a**) 250 °C, (**b**) 300 °C, and (**c**) 350 °C.

**Figure 8:**
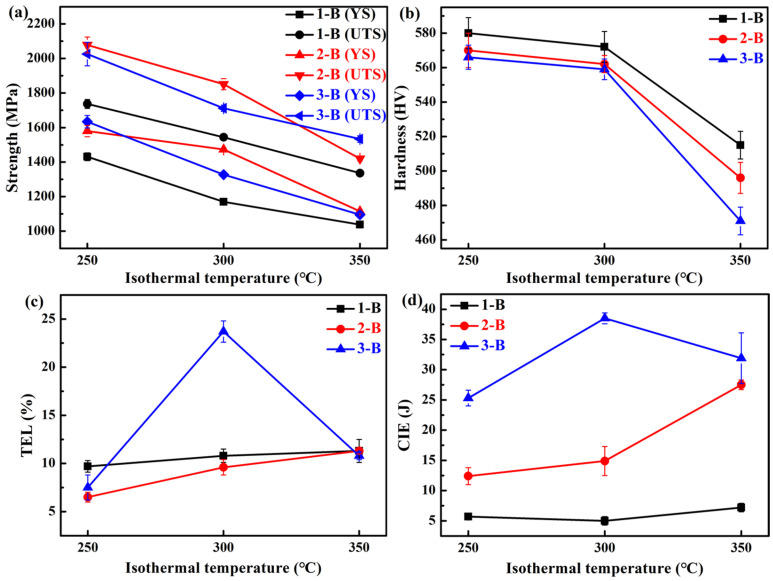
(**a**) YS and UTS, (**b**) hardness, (**c**) TEL, and (**d**) CIE with different isothermal temperatures of the investigated three steels.

Table 4 and Figure 7 show the mechanical properties and representative tensile curves of the investigated three steels treated by austempering processes, respectively. It can be seen that the YS, UTS, and hardness of the three steels gradually decrease with the increase in isothermal temperature (see Figure 8a,b). During isothermal holding at these temperatures, formed bainite could be tempered, which can induce softening by the occurrence of recovery [63]. In addition, an increase in the isothermal temperature can enhance the softening induced by tempering, thus resulting in a decrease in strength. In addition, necking appears in the tensile curves of the 1/2/3 B-300 and 1 B-350 (see Figure 7), whereas necking does not occur in the QT process (see Figure 5). The TEL of the No. 1 and No. 2 steels increases with the increase in isothermal temperature (see Figure 8c); however, for the No. 3 steel, when the temperature is 300 °C, the TEL reaches the maximum (23.7%). In Figure 8d, for the No. 1 steel, the CIE is low (<10 J). For the No. 2 steel, the CIE increases with the increase in isothermal temperature. For the No. 3 steel, when the temperature is 300 °C, the CIE reaches the maximum (38.5 J). The No. 3 steel treated by the austempering process exhibits a higher CIE compared with that of the other steels. On the whole, the strength and hardness of the three steels treated by the austempering process are lower than those by the QT process, while their ductility and toughness are superior.

#### 3.2.3. Mechanical Properties of the Three Steels Treated by QP Processes

**Figure 9:**
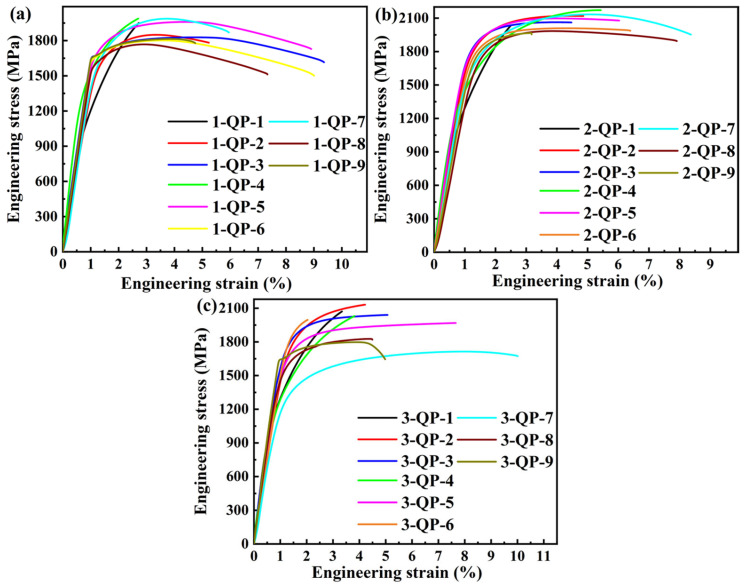
Representative engineering stress–strain curves of the investigated three steels treated by QP processes: (**a**) No. 1, (**b**) No. 2, and (**c**) No. 3.

**Figure 10:**
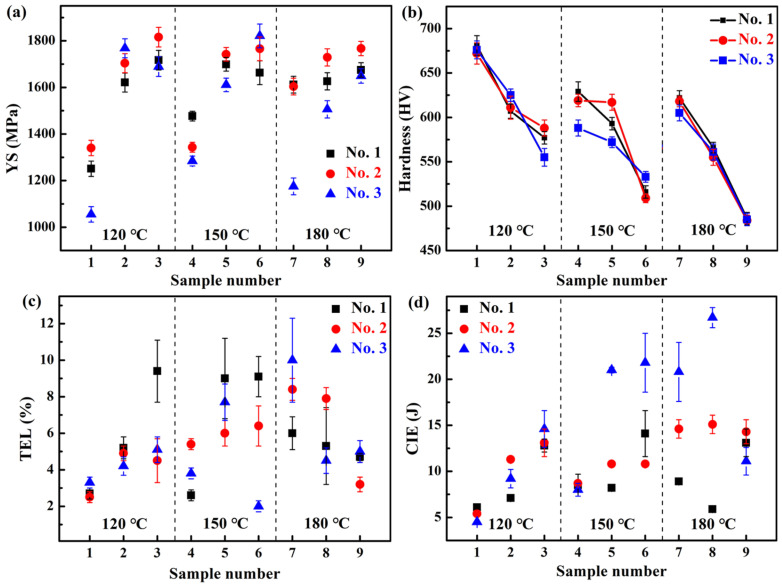
(**a**) YS, (**b**) hardness, (**c**) TEL, and (**d**) CIE with different quenching and partitioning temperatures of the investigated three steels.

Table 5 and Figure 9 show the mechanical properties and representative tensile curves of the investigated three steels treated by QP processes, respectively. It can be seen that for each quenching temperature of the three steels, the YS increases with the increase in partitioning temperature (see Figure 10a), which may be due to the C segregating at dislocations, locking the mobile dislocations and improving the YS [64], or carbide precipitation and bainite formation [52]. The maximum YS is 1821 MPa for the 3-QP-6 steel. The hardness for each quenching temperature of the three steels decreases with the increase in partitioning temperature (see Figure 10b), which is mainly related to the formed first martensite and bainite that are tempered during partitioning. The maximum hardness is 681 HV of the 1-QP-1 steel. For the TEL of the three steels treated by QP processes (see Figure 10c), they are lower than that by austempering processes and are essentially comparable to that by QT processes (<10%). In Figure 10d, the CIE generally exhibits an increasing trend with the rise in partitioning temperature for each quenching temperature of the three steels, and on the whole, the No. 3 steel shows a higher CIE compared with that of the other steels.

### 3.3. Microstructure of the Three Steels Treated by Different Heat Treatments

**Figure 11:**
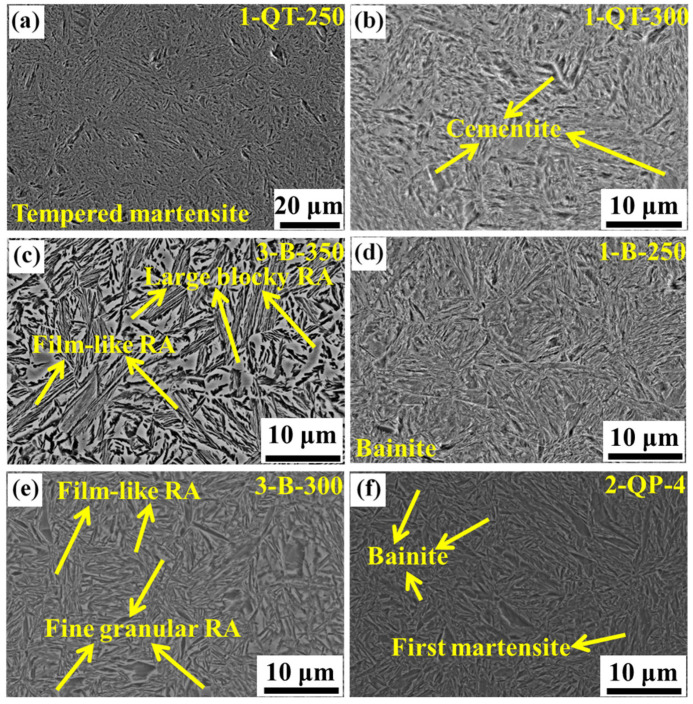
SEM micrographs show the representative microstructure of the three steels subjected by different heat treatments: (**a**) 1-QT-250, (**b**) 1-QT-300, (**c**) 3-B-350, (**d**) 1-B-250, (**e**) 3-B-300, and (**f**) 2-QP-4.

**Figure 12:**
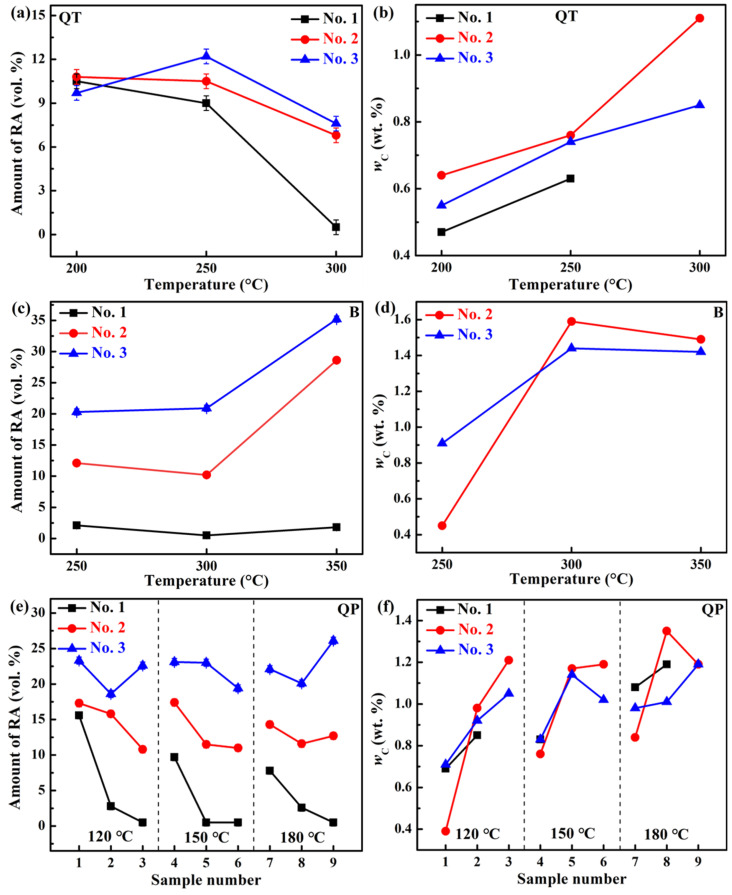
(**a**,**c**,**e**) show the RA content of the investigated three steels by different heat treatments, including QT, austempering, and QP, respectively. (**b**,**d**,**f**) show the *w*_C_ of the steels by different heat treatments.

The microstructure of the three steels treated by QT processes mainly consists of tempered martensite, and the representative microstructure is shown in Figure 11a. Figure 12a,b shows the *f*_RA_ and *w*_C_ as a function of tempering temperature of the three steels by XRD, respectively. It can be seen that overall, the *f*_RA_ decreases with the increase in tempering temperature of the three steels due to the decomposition of austenite, and the increase in Si content retards this decreasing trend. The No. 1 steel after tempering at 300 °C shows almost no RA, which indicates that the increase in Si content can improve the stability of RA. The *w*_C_ increases with the increase in tempering temperature of the three steels, which is due to the decrease in *f*_RA_ and the enhanced diffusion rate of carbon. In Figure 6a, the increase in strength with tempering temperature is primarily related to the decomposition of austenite into the BCC phase and carbides. For the QT process, the steels show low ductility and toughness (see Table 3). The tempering process plays a significant role in the cementite precipitation and its morphology. At low tempering temperature, the cementite precipitates have a stick-like structure, which leads to a deterioration in ductility and toughness [65] (see Figure 11b). Furthermore, in Figure 12b, the *w*_C_ is mostly below 1 wt. %, which suggests that the mechanical stability of RA is low, resulting in the low ductility and toughness.

For the austempering process, the *f*_RA_ shows an increasing trend with the increase in isothermal temperature for the three steels. For each isothermal temperature, quantitative analysis reveals that increasing the Si content from 0.75 wt. % to 2.72 wt. % leads to a significant increase in the *f*_RA_ under the B-300 condition, from 0.5 vol. % to 20.9 vol. %, which confirms that Si alloying plays a significant role in stabilizing austenite, as shown in Figure 12c. This indicates that Si can suppress the precipitation of cementite and slow down the bainitic transformation, and thus increase the *f*_RA_ [53]. In Figure 12d, the *w*_C_ exhibits an initial increase followed by stabilization with the increase in isothermal temperature, which is mainly due to accelerated bainitic transformation promoting carbon enrichment in the remaining and stabilized austenite [66]. For the 2/3 B-350 steels, their *f*_RA_ are more than 25 vol. %, resulting in the low hardness and strength (see Table 4), and thus they are not suitable for wear-resistant steel balls [67]. Generally, the hardness of the steel used for wear-resistant steel balls is 52–58 HRC. In Figure 11c, the microstructure of the 3 B-350 steel consists of bainitic ferrite, film-like RA, and large blocky RA. The large blocky RA can undergo rapid transformation to martensite during service (via the TRIP effect) due to the low mechanical stability, which has a detrimental effect on the ductility and toughness, thereby reducing its service life [68]. For the 1 B-250/300/350 steels, their *f*_RA_ are very low (see Figure 12c), and thus their ductility and toughness are inferior (see Table 4). The SEM micrograph in Figure 11d can provide supporting evidence for the low *f*_RA_. For the 3 B-300 steel, it exhibits a considerable amount of RA with high *w*_C_ (see Figure 12c,d), and the RA exhibits film-like and fine granular morphology (see Figure 11e), which indicate that the RA has a good mechanical stability and thus the steel has an excellent combination of ductility, toughness, strength and hardness (see Table 4), making it suitable for manufacturing wear-resistant steel balls.

For the QP process, the *f*_RA_ increases with the increase in Si content for each quenching temperature and partitioning temperature, and for the No. 1 and No. 2 steels, the *f*_RA_ decreases with the increase in partitioning temperature for each quenching temperature (see Figure 12e). For the No. 3 steel, the *f*_RA_ is ~20 vol. % for the selected quenching and partitioning temperatures. In Figure 12f, it can be seen that, on the whole, for each quenching temperature, the *w*_C_ increases with the increase in partitioning temperature of the three steels. When the partitioning temperature is 400 °C for the quenching temperatures of 150 °C and 180 °C, the hardness is low due to the tempering softening of the primary martensite, and thus, they are not suitable for wear-resistant steel balls (see Figure 10b). When the quenching temperature is 120 °C, the TEL is relatively low (see Figure 10c), which may be related to the high primary martensite content and thus the high strength and hardness and low ductility [69]. The representative microstructure of the steels treated by the QP process is shown in Figure 11f. The influence of QP process on the mechanical properties is associated with multiple factors, such as the volume fraction and mechanical stability of RA, the presence of secondary martensite, the bainite content, the amount of primary martensite and its tempering softening, as well as the coordinated deformation among the various microstructural constituents. Therefore, elucidating the relationship between the microstructure and the mechanical properties becomes more complex.

Table 1 shows that the increase in Si content can enhance the CE. A clear correlation exists between the CE, microstructural evolution, and the stability of RA. The progressive increase in Si content significantly suppresses the precipitation of cementite during both bainitic transformation and tempering processes. This suppression effect, enhanced by the higher CE, promotes carbon partitioning into the austenite, thereby increasing its stability. Consequently, as observed in Figure 12a,c,e, the *f*_RA_ generally increases with higher CE across all heat treatment conditions (QT, austempering, and QP). Furthermore, the mechanical stability of RA, reflected by its *w*_C_ shown in Figure 12b,d,f, is also enhanced at some specific temperatures, contributing to the improved combination of strength and toughness via the TRIP effect. Therefore, the CE, dominantly influenced by the Si content in this specific alloy design, serves as a critical indicator. It not only predicts hardenability but also directly correlates with key microstructural outcomes and abrasive wear resistance.

### 3.4. Analysis of Impact-Abrasive Wear Performance

The preceding section has illustrated the relationship between the microstructure and mechanical properties of the wear-resistant steels with different Si contents under various heat treatment processes. This section will focus on investigating the effect of Si content and heat treatments on the wear resistance. The optimum parameter of each heat treatment was selected. For QT, austempering, and QP processes, the QT-250, B-300, and QP-4 parameters were chosen, respectively, and the steels subjected to these heat treatments have excellent combined mechanical properties.

**Figure 13:**
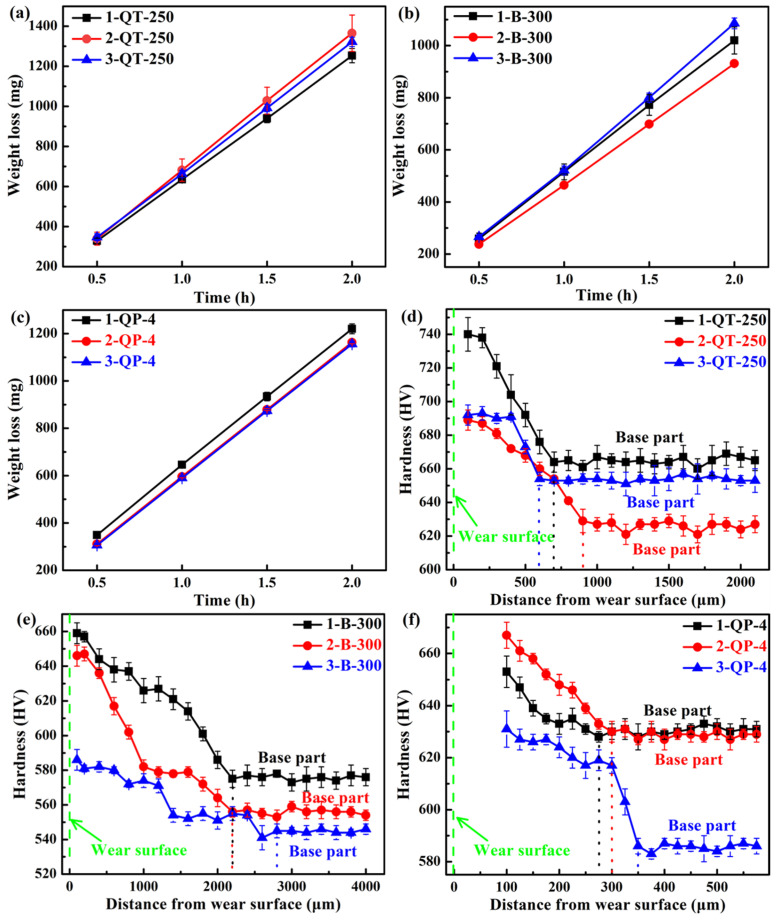
(**a**–**c**) Three-body impact-abrasive wear weight losses and (**d**–**f**) microhardness distributions after impact wear of the three steels subjected to different heat treatments.

The weight loss during impact-abrasive wear tests is plotted in Figure 13a–c. It can be seen that the weight loss increases with increasing testing time for the three selected heat treatments of the three steels. For the QT-250 process, the Si content does not have a significant impact on the weight loss, and the 1-QT-250 steel has the lowest weight loss (1253 mg) after a two-hour period of impact wear testing. In addition, the mass loss of the 1-QT-250 steel with the highest wear resistance was 8.2% lower than that of the 2-QT-250 with the lowest wear resistance after a two-hour impact wear test. The wear rates of the three steels are 627 mg/h, 683 mg/h, and 661 mg/h. The 1/2/3-QT-250 steels have similar microstructure and mechanical properties (see Table 3 and Figure 12a,b), and thus exhibit comparable wear resistance. For the B-300 process, the 2 B-300 steel demonstrates the best wear resistance, and its weight loss is 931 mg. Although its *f*_RA_ is moderate, the high *w*_C_ contributes to the high mechanical stability of RA. Furthermore, the YS and UTS of the 2 B-300 steel are higher than that of the 1/3 B-300 steels; however, the TEL and CIE of the 2 B-300 are lower than that of the 3 B-300 (see Figure 8), which may indicate that the strength has a more significant influence on wear resistance than ductility and toughness in terms of this heat treatment. The wear rates of the three steels are 510 mg/h, 466 mg/h, and 543 mg/h, which are much lower than those of the QT-250 process. For the QP-4 process, the No. 2 and No. 3 steels exhibit comparable wear resistance, with a nearly identical weight loss of 1156 mg after a two-hour impact wear test. In contrast, the No. 1 steel demonstrates inferior performance, which may be related to its low *f*_RA_. The wear rates of the three steels are 610 mg/h, 582 mg/h, and 578 mg/h, which were intermediate between those of the QT and austempering processes.

**Figure 14:**
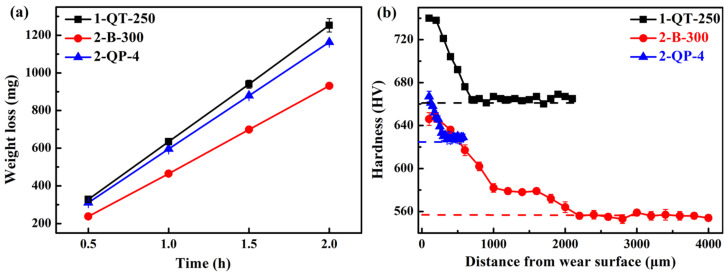
Plots of (**a**) mass loss versus wear time and (**b**) hardness distribution profiles after the impact wear tests for the steels with the best wear performance among the three processes.

The microhardness curves in the normal direction of the wear surface are shown in Figure 13d–f. It can be seen that the microhardness of all samples decreases with the increase in the distance below the wear surface and eventually approaches the matrix microhardness, indicating the work hardening capacities after the impact deformation. For the QT-250 process, the wear surface hardness of the 1-QT-250 steel increases by 12.1%, which is the highest, and the depth of the affected layer is approximately 700 μm. For the B-300 process, the 1 B-300 and 2 B-300 steels have the maximum increase in the wear surface hardness (~15%), and their depth of the affected layer is ~2200 μm. For the QP-4 process, the 2-QP-4 steel has the maximum increase in the wear surface hardness (~7.8%), and its depth of the affected layer is 300 μm. The 1/2 B-300 steels exhibit the highest work hardening capability, and their plastic deformation can penetrate more deeply into the matrix, thereby strengthening the strain-hardened layer affected by impact wear. During impact wear, the TRIP effect of RA can compensate for the effect of the initially low hardness of the 1/2 B-300 steels on the wear resistance. Furthermore, this effect can be amplified by increasing the impact energy [12]. The *f*_RA_ of the 1 B-300 steel is lower than that of the 2 B-300, and this may be the reason why the wear resistance of the 2 B-300 steel is better. Figure 14a,b summarizes the plots of mass loss versus wear time for the steels with the best wear performance among the three processes and the hardness distribution profiles after the impact wear tests, respectively. The deep-gradient-layer steel has the optimal impact-abrasive wear resistance [70].

Among the three steels in Figure 14, the 2 B-300 steel exhibits the lowest strength and hardness, yet the highest TEL and CIE. In addition, its *f*_RA_ is at a medium level, but the *w*_C_ is the highest. These suggest that excellent impact wear resistance could not be determined by a single outstanding mechanical property, but rather by the comprehensive performance of the steel. The overall mechanical properties are governed by the microstructure of steels. The *f*_RA_, *w*_C,_ and mechanical stability of RA have an important effect on the impact wear resistance [1]. Furthermore, the matrix microstructure serves as the essential foundation for improving this resistance. From Figure 13a–c, it can be seen that bainite provides the best impact wear resistance, outperforming the tempered martensite of QT steel and the mixed microstructure of QP steel, including primary martensite and bainite.

## 4. Discussion

### 4.1. Analysis of Impact-Abrasive Wear Surface Morphology

**Figure 15:**
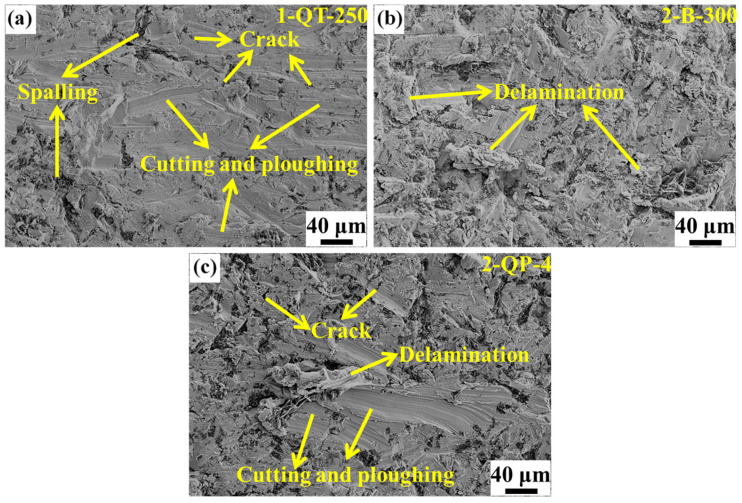
The wear surface morphologies of (**a**) 1-QT-250 steel, (**b**) 2 B-300 steel, and (**c**) 2-QP-4 steel after impact wear tests.

Figure 15 presents the wear surface morphologies of the three investigated steels, revealing a universally rough topography induced by the combined effect of impact loading and repeated rolling-sliding contact. Distinct damage mechanisms are evident across the different microstructures. Specifically, the surface of the 1-QT-250 steel (Figure 15a) is characterized by a high density of cracks, pronounced cutting and plowing grooves, and extensive spalling damage. The underlying wear process can be fundamentally described by a two-stage mechanism [1]. Initially, during the impact stage, abrasive particles are forcibly embedded into the material surface. Subsequently, in the rolling-sliding stage, a portion of these embedded particles is rolled out, leaving behind craters that act as stress concentration sites for crack initiation and propagation, while the remaining particles contribute to cutting and plowing wear. The coexistence of these mechanisms ultimately leads to the observed surface degradation. For the 1-QT-250 steel, the high primary hardness and strength, but low TEL and CIE, can give rise to the formation of cracks on the wear surface at the beginning of the wear process due to the accumulated plastic strain on the sample surface readily reaching a critical threshold under repeated impacts [1]. Prolonged wear can induce two distinct crack propagation pathways. In one mode, cracks extend horizontally along the surface, ultimately causing flake-like delamination damage. Alternatively, other cracks can directly penetrate to a certain depth and subsequently interconnect, leading to the removal of thick fragments and resulting in spalling damage. Moreover, the exposure of these spalled areas on the wear surface signifies a progressive worsening of the damage. Consequently, the 1-QT-250 steel has the worst wear resistance of the three selected steels. The 2 B-300 steel exhibits a marked reduction in cutting and plowing compared with that of the 1-QT-250 steel, and the delamination damage becomes the main feature of the wear surface (see Figure 15b). The reduction should be ascribed to the continuous TRIP effect, contributing to the release of the local stress concentration and surface hardening effect. The wear surface of the 2-QP-4 steel is shown in Figure 15c, and it mainly displays cracks, cutting, plowing, and delamination. The 2-QP-4 steel contains the highest amount of soft RA among the three steels, and thus it is more prone to plastic deformation under impact wear, exhibiting longer cutting and plowing grooves than the other two steels.

### 4.2. Effect of Residual Stress on the Wear Resistance

The residual stress has an important effect on the wear resistance of steels. The existence of a compressive stress state can delay the propagation of subsurface cracks and inhibit the delamination wear mechanism [71]. Conversely, the residual tensile stress can increase the speed of crack propagation, which decreases the wear resistance [72]. In terms of wear-resistant steel balls with a diameter of ~100 mm, during the forging and heat treatment process, residual stress can be generated due to the thermal and structural gradients, as well as phase transformation, which can affect the working life of the balls [73,74]. The thermal gradient arising during cooling induces differential contraction between the rapidly cooled surface and the slowly cooled interior. This incompatibility in deformation generates thermal stress, which is tensile at the surface. In contrast to thermal stress, transformation stress originates from the phase transformation of FCC into BCC during cooling. This stress is compressive in nature, developing as a result of the associated volumetric expansion. Therefore, understanding the contributions of these opposing stresses is critical to manipulating the final residual stress state and enabling the deliberate design of material properties [75].

**Figure 16:**
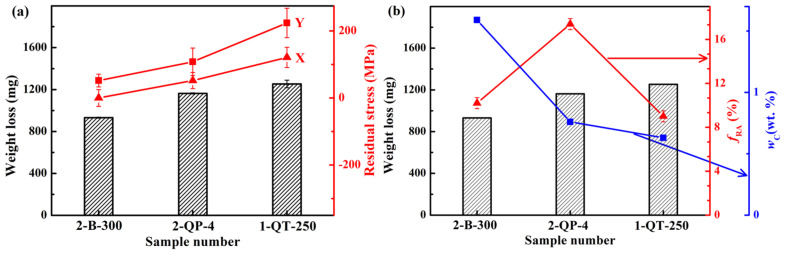
(**a**) Surface residual stresses in two directions of the 1-QT-250, 2 B-300, and 2-QP-4 steels, as well as the weight losses after impact-abrasive wear tests, and (**b**) weight losses after impact-abrasive wear tests and the fRA and wC of the 1-QT-250, 2 B-300, and 2-QP-4 steels.

Figure 16a shows the surface residual stresses in two directions of the 1-QT-250, 2 B-300, and 2-QP-4 steels, as well as the weight losses after impact-abrasive wear tests. It can be seen that the surface of the three steels is subjected to tensile stress, in which the residual stress in the Y direction at the surface of the 1-QT-250 steel is the maximum (224 MPa), and that of the 2 B-300 steel is the minimum (~50 MPa). In addition, the weight loss is found to be directly proportional to the magnitude of the residual tensile stress, which indicates that the surface residual stress plays an important role in the wear resistance. The 2 B-300 steel, which demonstrated the best wear resistance (lowest wear loss of 931 mg), possessed the smallest surface tensile residual stress. In contrast, the 1-QT-250 steel, with the highest wear loss (1253 mg), exhibited the highest surface tensile residual stress. This direct proportionality provides compelling quantitative evidence that minimizing surface tensile stress is a critical mechanism for enhancing impact-abrasive wear resistance. The high tensile stress facilitates crack initiation and propagation under impact loading, thereby accelerating material removal. The volume expansion of the surface phase transformation results in a reduction in the residual tensile stress caused by thermal stress. Furthermore, during martensitic or bainitic transformation, the different volume expansions and transformation kinetics, coupled with thermal stress generated during subsequent cooling between the surface and the core, lead to a complex interaction that ultimately determines the final stress state in the surface region [76]. To further clarify the interplay between microstructure and wear performance, Figure 16b introduces the relationship between wear mass loss and the *f*_RA_ and *w*_C_ of RA. This consolidated plot reveals a non-monotonic relationship. While a low *f*_RA_ (as in the 1-QT-250 steel) correlates with poor wear resistance, the highest *f*_RA_ (found in the 2-QP-4 steel) does not yield the optimal performance. The 2 B-300 steel occupies a favorable position, characterized by a moderate *f*_RA_ coupled with the highest *w*_C_ among the three samples. This combination indicates superior mechanical stability of its RA, which is crucial for a sustained and beneficial TRIP effect during impact-abrasive wear. The synergistic interpretation of both Figure 16a,b leads to a central conclusion of this study that the exceptional wear resistance of the 2 B-300 steel is not attributable to a single factor, but rather to the optimal synergy achieved through a specific microstructure (moderate *f*_RA_ with high stability) resulting in a beneficial residual stress state (low surface tensile stress) and high work-hardening capacity.

The significant impact of residual stress on the wear resistance of steels is well-established and is strongly corroborated by the findings of this work. As presented in Figure 16, a direct correlation is observed between the magnitude of surface tensile residual stress and the wear mass loss for the 2 B-300, 2-QP-4, and 1-QT-250 steels. Specifically, the 2 B-300 steel, which exhibited the lowest wear loss, also possessed the smallest surface tensile residual stress (approximately 50 MPa). This finding aligns with the results of Tomaz et al. [71], who demonstrated that compressive residual stress from shot peening could reduce the sliding wear rate of AISI 4340 steel by approximately fifty percent. Both studies underscore that mitigating tensile or introducing compressive stresses at the surface is a critical mechanism for enhancing wear resistance by suppressing crack propagation. The final residual stress state in our steels is a complex outcome of competing thermal gradients (generating tensile stress) and phase transformation-induced volumetric expansion (generating compressive stress). Camurri et al. [73] modeled the temperature distribution, phase transformation, and residual stress induced during the heat treatment of grinding balls with diameters of 76 mm and 127 mm, and this model enabled the optimization of heat treatment processes and the resultant residual stress. Our results, showing varied stress states for different microstructures (tempered martensite vs. bainite), suggest that the kinetics of martensitic and bainitic transformations under our specific cooling conditions played a decisive role. This is consistent with the work of Lee et al. [75], who linked increased surface temperature and cooling rate during flame hardening of 12Cr steel to higher tensile residual stress, which was subsequently relaxed by crack propagation. Furthermore, the influence of microalloying elements on stress evolution, as reported by Dong et al. [77], who investigated the effect of cooling rate on microstructure, hardness and residual stress of 0.28C-0.22Ti wear-resistant steel, and demonstrated that the range of the residual stress caused by the hard particles decreased with the decrease in cooling rate, indicating that the TiC particles significantly contributed to the residual stress in the high-titanium steels, provides a parallel to the significant role that our alloy design (particularly Si content) played in determining the phase transformation behavior and resultant stress. The ability to tailor the residual stress through heat treatment is evident from the work of Lu et al. [74]. They investigated the influence of the interrupted quenching process on residual stress and mechanical properties of martensitic wear-resistant steel, and found that this process could effectively manage residual stress. While our QP and austempering processes are distinct, they share the common principle of using interrupted cooling to manipulate the microstructure and thus the residual stress.

In conclusion, while this study has firmly established the critical relationship between the final residual stress, microstructure, and wear performance in our Si-alloyed steels, the precise temporal evolution and interaction of thermal and phase transformation stresses (particularly from martensitic and bainitic transformations) remain a complex subject. Therefore, a detailed investigation into the underlying mechanisms governing this evolution is identified as a key objective for future research.

## 5. Conclusions

This work systematically investigated the effect of Si content (0.75, 1.34, and 2.72 wt. %) on the impact-abrasive wear behavior of medium-carbon low-alloy steels subjected to QT, austempering, and QP processes. The key findings, supported by quantitative data, are synthesized as follows:

(1) The study establishes a clear quantitative relationship between Si content, heat treatment, and resulting microstructure. Si addition significantly enhanced the *f*_RA_ and *w*_C_ of RA. Under the B-300 austempering condition, increasing Si from 0.75 wt. % to 2.72 wt. % raised the *f*_RA_ from 0.5 vol. % to 20.9 vol. %. Austempering generally provides the most balanced mechanical properties. Specifically, the 2 B-300 steel (1.34 wt. % Si) exhibited an excellent combination of strength (YS: 1473 MPa, UTS: 1851 MPa), ductility (TEL: 9.6%), and toughness (CIE: 14.9 J), attributable to the stable and film-like RA.

(2) A direct correlation was established between microstructure, residual stress, and wear resistance. The 2 B-300 steel demonstrated superior wear resistance, with the lowest cumulative mass loss of 931 mg after 2 h testing (wear rate: 466 mg/h), significantly outperforming the best QT (1-QT-250, 1253 mg, 627 mg/h) and QP (2-QP-4, 1156 mg, 578 mg/h) samples. This superiority is attributed to its high work-hardening capacity, evidenced by a ~15% increase in surface hardness and a deep strain-hardened layer extending approximately 2200 µm beneath the wear surface, coupled with a beneficial residual stress state.

(3) The surface residual stress was identified as a critical factor governing wear resistance. A strong quantitative correlation was observed; higher surface tensile residual stress correlated with greater wear loss. The 2 B-300 steel possessed the lowest surface tensile residual stress (~50 MPa), while the less wear-resistant 1-QT-250 steel exhibited the highest (224 MPa). This underscores the importance of inducing compressive stresses or minimizing tensile stresses at the surface.

(4) The results provide clear and quantitative guidance for designing wear-resistant components such as grinding balls and mining equipment. The optimal performance was achieved not with the highest Si content, but with a specific balance, a Si content of approximately 1.3 wt. % combined with an austempering treatment at 300 °C (B-300 process). This combination yields a microstructure with an optimal balance of high-strength bainitic ferrite and mechanically stable RA, leading to exceptional impact-abrasive wear resistance mediated by a pronounced TRIP effect, deep work-hardening, and low residual tensile stress.

(5) While this study identifies the optimal Si content and heat treatment, the precise mechanisms behind the superior performance of the 1.34 wt. % Si composition, particularly its interaction with phase transformation kinetics and residual stress evolution, warrants deeper investigation. Future work will focus on elucidating these mechanisms to further refine alloy design strategies.

## Figures and Tables

**Table 1:** Chemical compositions and CE of the three investigated steels (wt. %).

Sample Number	C	Si	Mn	Cr	Ti	O	P	S	Fe	CE
1	0.67	0.75	0.78	0.80	0.057	0.0017	0.0041	0.0032	96.9340	1.085
2	0.64	1.34	0.80	0.72	0.040	0.0021	0.0035	0.0035	96.4509	1.141
3	0.65	2.72	0.80	0.73	0.058	0.0019	0.0038	0.0031	95.0332	1.383

**Table 2:** Parameters of QP processes and their designated numbers.

	QP-1	QP-2	QP-3	QP-4	QP-5	QP-6	QP-7	QP-8	QP-9
Quenching temperature (°C)	120	120	120	150	150	150	180	180	180
Partitioning temperature (°C)	300	350	400	300	350	400	300	350	400

**Table 3:** Mechanical properties of the investigated three steels treated by QT processes.

Number	YS (MPa)	UTS (MPa)	TEL (%)	Hardness (HV)	CIE (J)	E (GPa)
1-QT-200	1357 ± 33	1904 ± 70	2.6 ± 0.3	692 ± 9	4.8 ± 0.4	209 ± 4
1-QT-250	1633 ± 41	2018 ± 61	3.3 ± 0.4	660 ± 6	5.4 ± 0.2	206 ± 3
1-QT-300	1717 ± 42	2017 ± 67	4.1 ± 1.1	582 ± 5	4.1 ± 0.2	202 ± 8
2-QT-200	1378 ± 21	1842 ± 58	2.3 ± 0.3	679 ± 7	3.9 ± 0.5	207 ± 4
2-QT-250	1551 ± 29	2038 ± 67	4.5 ± 0.2	635 ± 9	6.1 ± 0.4	195 ± 8
2-QT-300	1814 ± 51	2002 ± 64	5.4 ± 1.1	598 ± 6	6.5 ± 0.4	190 ± 12
3-QT-200	1562 ± 36	1814 ± 69	3.3 ± 0.2	676 ± 9	6.4 ± 0.6	207 ± 4
3-QT-250	1669 ± 37	1989 ± 48	4.5 ± 0.3	654 ± 6	6.1 ± 0.4	209 ± 6
3-QT-300	1725 ± 31	1939 ± 62	4.4 ± 0.2	641 ± 5	9.9 ± 0.7	210 ± 10

**Table 4:** Mechanical properties of the investigated three steels treated by austempering processes.

Number	YS (MPa)	UTS (MPa)	TEL (%)	Hardness (HV)	CIE (J)	E (GPa)
1 B-250	1431 ± 23	1737 ± 26	9.7 ± 0.6	580 ± 9	5.7 ± 0.5	207 ± 4
1 B-300	1170 ± 9	1544 ± 16	10.8 ± 0.7	572 ± 9	5.0 ± 0.7	202 ± 6
1 B-350	1038 ± 6	1336 ± 14	11.3 ± 1.2	515 ± 8	7.2 ± 0.7	195 ± 8
2 B-250	1580 ± 33	2078 ± 46	6.5 ± 0.5	570 ± 10	12.4 ± 1.4	202 ± 4
2 B-300	1473 ± 8	1851 ± 32	9.6 ± 0.8	562 ± 5	14.9 ± 2.4	201 ± 5
2 B-350	1115 ± 9	1420 ± 14	11.3 ± 0.3	496 ± 9	27.5 ± 0.8	200 ± 2
3 B-250	1634 ± 36	2026 ± 68	7.5 ± 1.3	566 ± 7	25.3 ± 1.3	204 ± 3
3 B-300	1327 ± 13	1712 ± 18	23.7 ± 1.1	559 ± 6	38.5 ± 0.9	201 ± 4
3 B-350	1095 ± 12	1534 ± 19	10.8 ± 0.4	471 ± 8	31.9 ± 4.2	200 ± 1

**Table 5:** Mechanical properties of the investigated three steels treated by QP processes.

Number	YS (MPa)	UTS (MPa)	TEL (%)	Hardness (HV)	CIE (J)	E (GPa)
1-QP-1	1251 ± 33	1986 ± 20	2.7 ± 0.3	681 ± 11	6.1 ± 0.2	205 ± 5
1-QP-2	1621 ± 41	1789 ± 60	5.2 ± 0.6	607 ± 8	7.1 ± 0.2	201 ± 3
1-QP-3	1717 ± 42	1824 ± 70	9.4 ± 1.7	577 ± 7	12.8 ± 0.7	205 ± 3
1-QP-4	1477 ± 21	1975 ± 14	2.6 ± 0.3	629 ± 11	8.5 ± 1.2	208 ± 5
1-QP-5	1699 ± 29	2006 ± 67	9.0 ± 2.2	593 ± 7	8.2 ± 0.1	204 ± 3
1-QP-6	1663 ± 51	1814 ± 64	9.1 ± 1.1	516 ± 7	14.1 ± 2.5	203 ± 3
1-QP-7	1612 ± 36	2076 ± 69	6.0 ± 0.9	622 ± 8	8.9 ± 0.2	200 ± 4
1-QP-8	1626 ± 37	1996 ± 146	5.3 ± 2.1	566 ± 6	5.9 ± 0.1	204 ± 3
1-QP-9	1675 ± 31	1811 ± 32	4.7 ± 0.2	486 ± 7	13.1 ± 1.5	203 ± 3
2-QP-1	1340 ± 33	2033 ± 70	2.5 ± 0.3	672 ± 12	5.4 ± 0.1	205 ± 6
2-QP-2	1704 ± 41	2142 ± 33	4.9 ± 0.4	611 ± 13	11.3 ± 0.3	206 ± 3
2-QP-3	1816 ± 42	2077 ± 64	4.5 ± 1.2	588 ± 9	13.1 ± 1.5	205 ± 3
2-QP-4	1343 ± 21	2171 ± 58	5.4 ± 0.3	619 ± 7	8.7 ± 0.1	204 ± 5
2-QP-5	1742 ± 29	2097 ± 67	6.0 ± 0.7	617 ± 9	10.8 ± 0.2	210 ± 3
2-QP-6	1766 ± 51	2022 ± 64	6.4 ± 1.1	509 ± 5	10.8 ± 0.2	204 ± 3
2-QP-7	1604 ± 36	2134 ± 69	8.4 ± 0.6	618 ± 6	14.6 ± 1.0	208 ± 4
2-QP-8	1729 ± 37	1983 ± 48	7.9 ± 0.6	555 ± 9	15.1 ± 1.0	202 ± 4
2-QP-9	1767 ± 31	1966 ± 81	3.2 ± 0.4	484 ± 6	14.3 ± 1.3	201 ± 5
3-QP-1	1055 ± 33	2079 ± 43	3.3 ± 0.3	676 ± 10	4.5 ± 0.1	205 ± 2
3-QP-2	1768 ± 41	2107 ± 81	4.2 ± 0.5	625 ± 7	9.2 ± 1.0	204 ± 5
3-QP-3	1689 ± 42	2032 ± 77	5.1 ± 0.7	555 ± 10	14.6 ± 2.0	204 ± 5
3-QP-4	1284 ± 21	2044 ± 58	3.8 ± 0.3	588 ± 9	8.0 ± 0.7	207 ± 3
3-QP-5	1611 ± 29	1982 ± 67	7.7 ± 1.0	572 ± 6	21.0 ± 0.3	208 ± 1
3-QP-6	1821 ± 51	2002 ± 64	2.0 ± 0.3	533 ± 6	21.8 ± 3.2	205 ± 2
3-QP-7	1175 ± 36	1736 ± 69	10.0 ± 2.3	605 ± 9	20.8 ± 3.2	200 ± 4
3-QP-8	1506 ± 37	1814 ± 48	4.5 ± 0.7	561 ± 9	26.7 ± 1.1	206 ± 1
3-QP-9	1649 ± 31	1874 ± 62	5.0 ± 0.6	485 ± 7	11.1 ± 1.5	204 ± 2

## Data Availability

The original contributions presented in this study are included in the article. Further inquiries can be directed to the corresponding author.
